# Reinnervation of Bilateral Posterior Cricoarytenoid Muscles Using the Left Phrenic Nerve in Patients with Bilateral Vocal Fold Paralysis

**DOI:** 10.1371/journal.pone.0077233

**Published:** 2013-10-02

**Authors:** Meng Li, Shicai Chen, Hongliang Zheng, Donghui Chen, Minhui Zhu, Wei Wang, Fei Liu, Caiyun Zhang

**Affiliations:** Department of Otolaryngology-Head & Neck Surgery, Changhai Hospital, The Second Military Medical University, Shanghai, People’s Republic of China; Harvard Medical School, United States of America

## Abstract

**Objective:**

To evaluate the feasibility, effectiveness, and safety of reinnervation of the bilateral posterior cricoarytenoid (PCA) muscles using the left phrenic nerve in patients with bilateral vocal fold paralysis.

**Methods:**

Forty-four patients with bilateral vocal fold paralysis who underwent reinnervation of the bilateral PCA muscles using the left phrenic nerve were enrolled in this study. Videostroboscopy, perceptual evaluation, acoustic analysis, maximum phonation time, pulmonary function testing, and laryngeal electromyography were performed preoperatively and postoperatively. Patients were followed-up for at least 1 year after surgery.

**Results:**

Videostroboscopy showed that within 1 year after reinnervation, abductive movement could be observed in the left vocal folds of 87% of patients and the right vocal folds of 72% of patients. Abductive excursion on the left side was significantly larger than that on the right side (*P* < 0.05); most of the vocal function parameters were improved postoperatively compared with the preoperative parameters, albeit without a significant difference (*P* > 0.05). No patients developed immediate dyspnea after surgery, and the pulmonary function parameters recovered to normal reference value levels within 1 year. Postoperative laryngeal electromyography confirmed successful reinnervation of the bilateral PCA muscles. Eighty-seven percent of patients in this series were decannulated and did not show obvious dyspnea after physical activity. Those who were decannulated after subsequent arytenoidectomy were not included in calculating the success rate of decannulation.

**Conclusions:**

Reinnervation of the bilateral PCA muscles using the left phrenic nerve can restore inspiratory vocal fold abduction to a physiologically satisfactory extent while preserving phonatory function at the preoperative level without evident morbidity.

## Introduction

Bilateral vocal fold paralysis (BVFP), which is caused by injury to the bilateral recurrent laryngeal nerves (RLNs), is a potentially fatal condition that usually presents as progressive airway compromise with or without obvious voice changes. The traditional treatments for BVFP include cordectomy, laser posterior cordectomy, arytenoidectomy and laterofixation of the vocal folds, tracheotomy, and tracheostomy. All are effective in improving airway compromise at the cost of possible deterioration of the voice and aspiration. Hence, an ideal treatment should aim at re-establishment of physiologic inspiratory abductive movement and phonatory adductive movement of the bilateral vocal folds with preservation of a normal or near-normal voice quality, which would theoretically be attained by selective reinnervation of the bilateral posterior cricoarytenoid (PCA) muscles.

The nerve muscle pedicle (NMP) technique which was popularized by Tucker [1] and used in the treatment of vocal fold paralysis in earlier studies, has been abandoned since other surgeons were unable to use the technique satisfactorily [2-4]. Electrophysiological, histological, and anatomical studies have shown that the phrenic nerve is the optimal transfer nerve for reinnervation of the PCA muscle in treating patients with BVFP [5-7]. Numerous animal experiments have proven the feasibility of reinnervation of the PCA muscle to reacquire vocal fold abduction using the phrenic nerve as a donor nerve [6,7], while reports on human beings in this area are relatively limited. This may be partly due to the variability and complexity of the nerve supply of the human laryngeal muscle [8,9]. Crumley first introduced a split-phrenic nerve graft procedure, and the results in animal experiments were encouraging [4,10]. However, he clinically applied phrenic nerve implantation technique in a series of five patients and failed to re-establish visible inspiratory vocal fold abduction [5]. In 2002, we reported a clinical series of six patients with BVFP, among whom one PCA muscle was reinnervated using one single phrenic nerve and the other using an ansa-NMP transfer. Five patients showed inspiratory abductive motion on the phrenic transfer side, while no patients showed motion on the NMP implantation side [11]. Marie et al. also reported a series of 12 patients with BVFP who underwent selective reinnervation of the bilateral PCA muscles with one right upper phrenic nerve root using an interposition-free nerve graft and the adductor muscles with thyrohyoid branches of the hypoglossal nerve. Three of six evaluable patients showed active arytenoid abduction [12].

In the present study, 44 patients underwent selective reinnervation of the bilateral PCA muscles with the left phrenic nerve. The feasibility, effectiveness, and safety of this procedure were evaluated.

## Materials and Methods

### Patient characteristics

This study was approved by the Institutional Review Board of Second Military Medical University. We reviewed the charts of 44 patients (13 males and 31 females; mean age, 41.3 years; range, 17–59 years) with BVFP from 1 to 15 months; the average interval was 6.2 months. Among them, 18 had undergone tracheotomy while the other 26 had not. Manifestations included variable inspiratory dyspnea (I°-III°), hoarseness, and aspiration. Laryngoscopy revealed that the bilateral vocal folds were fixed in the midline or paramedian position. Laryngeal electromyography (EMG) indicated neurogenic injury of the bilateral RLNs. Arytenoid palpation and arytenoid CT were also performed before reinnervation surgery to exclude vocal fold immobilization caused by cricoarytenoid joint dislocation, trauma, or inflammation. The inclusion criteria in the present study is BVFP patients with a definite etiology of bilateral recurrent laryngeal nerve lesions caused by thyroid surgery or neck trauma; with age no more than 59 years old and the denervation course less than 15 months. If RLN injury was caused by thyroid surgery and the duration of lesion was less than six months, and surgeons of primary thyroid surgery suggested that RLNs might have been transected, and LEMG indicated severe neurogenic injury of bilateral RLNs, bilateral RLNs were initially explored during the surgery. Phrenic nerve transfer surgery was performed only on those whose bilateral RLNs were confirmed to be transected. If RLN lesion was caused by neck trauma, or thyroid surgery –related RLN lesion with a denervation course more than 6 months, then phrenic nerve transfer surgery was performed directly.

The exclusion criteria in the present study is: i). Bilateral vocal fold mobility impairment was caused by etiologies other than RLN injury, for example, cricoarytenoid joint dislocation or injury, cricoarytenoid arthritis, idiopathic vocal fold paralysis, et al; ii). Patients who had pulmonary diseases such as chest or lung trauma, emphysema, and chronic obstructive pulmonary disease which could possibly affect the pulmonary function of the patients; iii). The injury site was near the distal terminal end of the RLN or the distal branches of RLN were injured, precluding nerve anastomosis.

In this series of cases, Bilateral RLNs were confirmed to be completely transected during exploratory surgery in 15 BVFP patients with a denervation duration less than 3 months and 3 cases with a denervation duration 3-6 months, and phrenic nerve transfer surgery was then performed. For 12 patients whose RLN lesions were caused by neck trauma, at least 6 months were waited so as to allow for possible spontaneous recovery before we perform the surgery. And for another 14 BVFP patients caused by thyroid surgery whose denervation duration was more than 6 months, phrenic nerve transfer surgery was directly performed. All clinical investigations were conducted according to the principles expressed in the Declaration of Helsinki. Informed consent was obtained from all adult patients and from guardians for all minor patients involved in the present study. The consents were written and the ethics committees approved the consent procedure. Patients were followed up for at least 1 year after phrenic nerve transfer surgery.

### Operation procedure

**Figure 1:**
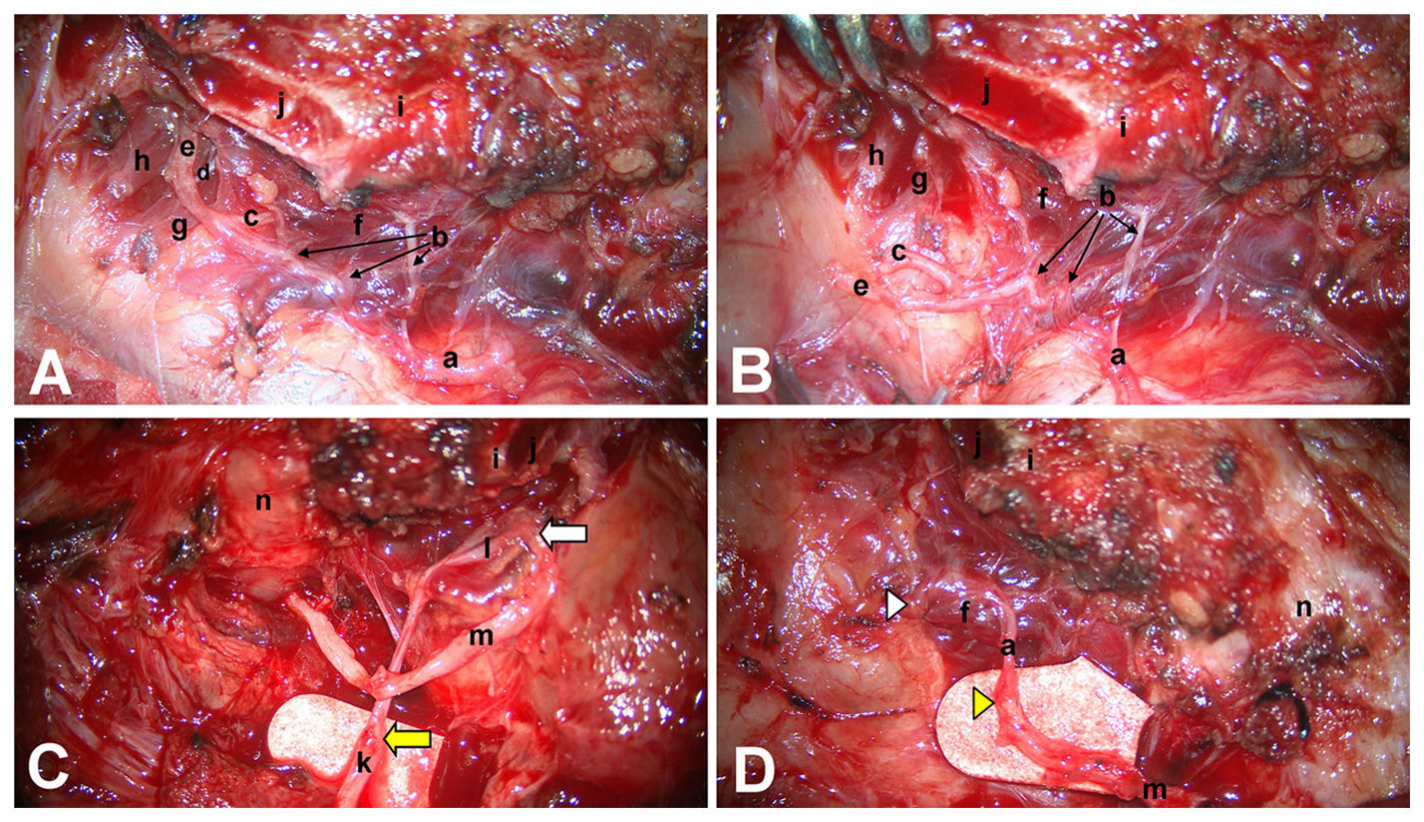
Intraoperative image of reinnervation of the bilateral PCA muscles using the left phrenic nerve. The posteroinferior portion of the thyroid lamina was partially removed. All adductor branches of the RLN, including the thyroarytenoid branch, lateral cricoarytenoid branch, interarytenoid branch, and three abductor branches, were well exposed (A). All adductor branches of the RLN were sectioned, and three abductor branches were well protected (B). The distal stump of the left RLN was anastomosed to the left phrenic nerve. A free nerve graft was used as a bridge between the adductor trunk of the left intralaryngeal RLN and distal stump of the right RLN through a retrolaryngeal or retrotracheal tunnel (C). The proximal ends of the adductor branches of the right intralaryngeal RLN were implanted into the right PCA muscle belly by suturing the nerve epineurium with the PCA myolemma (D). a: Recurrent laryngeal nerve (RLN), b: Abductor branches; c: Interarytenoid branch; d: .Lateral cricoarytenoid branch; e: Thyroarytenoid branch; f: Posterior cricoarytenoid muscle; g: Lateral cricoarytenoid muscle; h: Thyroarytenoid muscle; i: Inferior horn of thyroid cartilage; j: Section of thyroid lamina; k: Phrenic nerve; l: Adductor trunk of the RLN; m: Free nerve graft; n: Trachea. The yellow arrow in Figure 1C: the anastomotic site between phrenic nerve and the distal end of left RLN trunk; The white arrow in Figure 1C: the anastomotic site between adductor trunk of left RLN and free nerve graft; The yellow arrow head in Figure 1D: the anastomotic site between free nerve graft and the distal end of right RLN trunk; The white arrow head in Figure 1D: the implantation site of the proximal ends of adductor branches of the right RLN.

For patients who had not yet undergone tracheotomy, tracheotomy was first performed under local anesthesia. General anesthesia was then induced through the tracheotomy incision, direct laryngoscopy was performed, and the bilateral arytenoids were palpated to determine that they were passively mobile. If the arytenoids were found to be fixed, arytenoidectomy was adopted instead of phrenic nerve transfer because reinnervation could not be successful in such a case. A horizontal oblique incision was made at the original incision of the primary surgery or approximately 2 cm above the tracheotomy incision. The fascia between the strap muscles and the sternomastoid muscle was opened on the left side. The lower part of the carotid sheath was exposed, and the internal jugular vein was pulled laterally with a hook. The phrenic nerve was usually found deep under the transverse cervical artery. If the accessory phrenic nerve was found, it was kept intact. The phrenic nerve was cut off at the clavicle level. The cricopharyngeal muscle was incised on the left side, and the intralaryngeal segment of the RLN was dissected and exposed at the posterior-anterior part of the cricothyroid joint. The RLN was then traced proximally until the injured site of the RLN was found. The distal end of the RLN was prepared with resection of scarred tissue back to the nerve fascicles. The posteroinferior portion of the thyroid lamina was partially removed to allow for exposure of the intralaryngeal branches of the RLN (Figure 1A). All adductor branches of the RLN, including the thyroarytenoid branch, lateral cricoarytenoid branch, and interarytenoid branch, were sectioned, and injury to abductor branches was avoided (Figure 1B). The right RLN was exposed, and branches of the intralaryngeal segment were managed in the same way. The proximal end of the left phrenic nerve was transposed superiorly, to be anastomosed to the distal end of the left RLN using three to five epineurial 11-0 nylon sutures under a surgical microscope. The intralaryngeal adductor trunk of the left RLN was sectioned and sutured to a free nerve graft (a branch of the cervical plexus, usually the great auricular nerve or transverse nerve), 5 to 6 cm in length (Figure 1C), which was then passed contralaterally through a retrolaryngeal or retrotracheal tunnel and anastomosed to the distal end of the right RLN. The adductor branches of the right RLN were sectioned intralaryngeally, and their proximal ends were implanted to the right PCA muscle by suturing the adductor trunk epineurium with the PCA myolemma using a single 11-0 nylon suture (Figure 1D). Finally, the wound was closed in layers.

### Evaluation of surgery effect

The effect of the surgery was evaluated in four aspects. The preoperative assessment was performed directly before the operation, and the postoperative voice assessment, videostroboscopy, and pulmonary function test were performed at 2, 3, 4, 6 12 months after surgery in all cases. EMG was performed 1 year after surgery. Data obtained at 12 months postoperatively (with the exception of pulmonary function test) were used for statistical anaysis.

#### Videostroboscopy

All patients were examined using a videostroboscope (EndoSTROB DX, XION, Germany) at rest and after adequate exercise. Patients were observed during deep respiration and during “eee” phonation at a comfortable loudness and pitch as long as possible, and dynamic videos were recorded. Three experienced laryngologists who had not performed any operations reviewed all of the videos and scored the bilateral vocal fold movement during respiration and glottal closure during phonation. The videos were randomized and blinded to the reviewers. Our videostroboscopic rating method, a combination of the methods described by Lorenz [13], Rosen [14], and the present authors, included bilateral vocal fold abduction movement (0, no abduction; 1, slight abduction; 2, moderate abduction; 3, severe abduction), glottal closure during phonation (0, complete; 1, slightly incomplete; 2, moderately incomplete; 3, severely incomplete), and vocal fold edge of bilateral vocal folds (0, straight; 1, mildly bowing; 2, moderately bowing; 3, severely bowing). Consensus on the visual appearance of the larynx was reached among the reviewers.

#### Vocal function assessment

Vocal function assessment included perceptual evaluation, acoustic analysis, and maximum phonation time (MPT). Preoperative and postoperative voice samples, which contained the sustained vowels /a/ and connected speech samples, were subjected to perceptual evaluation and acoustic analysis. The recording equipment comprised a digital audiotape recorder and a dynamic microphone (Tiger Electronics Inc., North Reading, MA). Five laryngologists performed voice perceptual evaluation using a perceptual rating scale for voice quality and characteristics. The ratings were accomplished in a blinded fashion with patient voice samples arranged in a random manner. The listeners were asked to grade connected speech samples for overall grade, roughness, breathiness, asthenia, and strain (GRBAS). This perceptual scale allowed each listener to rate the voice quality on a scale (0, normal; 1, mild; 2, moderate; 3, severe) for each of the above parameters. The values were averaged among the five listeners [15].

The acoustic parameters of the sustained vowel /a/ were evaluated by Praat software (version 5.1.12). The acoustic parameters involved the mean noise-to-harmonics ratio (NHR) and measures of phonatory stability, namely jitter (local) and shimmer (local). MPT (the duration of sustained phonation of the vowel /a/ after maximum inspiration) was measured preoperatively and postoperatively. MPT is generally thought to be an index of glottal efficiency [15].

#### Decannulation rate

For those patients whose vocal folds showed abductory movement, the tracheal cannula was plugged for at least 48 h, and then decannulated only in the cases who showed no dyspnea. The success rate of this series is defined as succcessful decannulation after reinnervation procedure without subsequent revision operation such as arytenoidectomy.

#### Diaphragmatic movement and pulmonary function testing

Bilateral diaphragmatic movement was observed by chest X-ray. Pulmonary functional parameters, including vital capacity (VC), forced vital capacity (FVC), forced expiratory volume in 1 second (FEV1), and maximal inspiratory pressure (PImax) were obtained by pulmonary function testing (Vmax, SensorMedics, US). Pulmonary function tests were performed on patients who were wearing tracheal cannula as described by Misiolek [16].

#### Laryngeal EMG

A four-channel electromyograph and concentric needle electrodes (Dantec Counterpoint, Copenhagen, Denmark) were used for the EMG recording. The direction and depth of insertion of the electrodes were adjusted to test for the proper needle position. Electromyographic activities of the bilateral TA and PCA muscles were recorded simultaneously during the following two stages: 1) when the patients breathed quietly with relaxation of the body and 2) when the patients breathed deeply at rest and after physical activity, and sustained the vowel /eee/ with the greatest exertion. One board-certified otolaryngologist performed the EMG, and a neurologist operated the EMG machine and interpreted the EMG results. The neurologist rated the motor-unit recruitment (MUR) using the following scale: 0, full interference; 1, mixed interference; 2, simple interference; and 3, without motor unit potential. They then judged whether misdirected regeneration electric activities were present [15].

### Statistical analysis

The perceptual evaluation data are presented as medians (lower and upper quartiles). The acoustic analysis data, MPT values, and pulmonary functional parameters are presented as $\bar{x}$ ± s. Statistical differences between preoperative and postoperative data of videostroboscopy and EMG were analyzed using the chi-square ($\begin{array}{c} \chi_{CMH}{}^{{\text{2}}_{}} \end{array}$) test. Statistical differences between preoperative and postoperative perceptual evaluations were identified using Wilcoxon’s signed-rank test, statistical differences between the preoperative and postoperative acoustic analysis and MPT data were analyzed by paired *t*-test, and statistical differences between the pulmonary functional parameters in different postoperative intervals were analyzed by ANOVA and the least significant difference test. All analyses were conducted using SAS software (version 9.1.3). A *P* value of <0.05 was deemed to indicate statistical significance.

## Results

All patients presented varying degrees of aspiration after surgery, which disappeared about 2 to 8 weeks postoperatively, and normal deglutition function was achieved. A local hematoma and chylous fistula developed in one patient each and were resolved with appropriate treatment within 1 to 2 weeks. Recovery of unilateral or bilateral vocal fold abduction was achieved in 41 cases, later deterioration was observed in 3 of them. The rest 38 cases (87% of this series) were successfully decannulated 3 to 8 months after surgery and could perform considerable physical activity. Those who were decannulated after subsequent arytenoidectomy were not included in calculating the success rate of decannulation. Evaluation results of the surgery effects are as follows.

### Videostroboscopic findings

**Figure 2:**
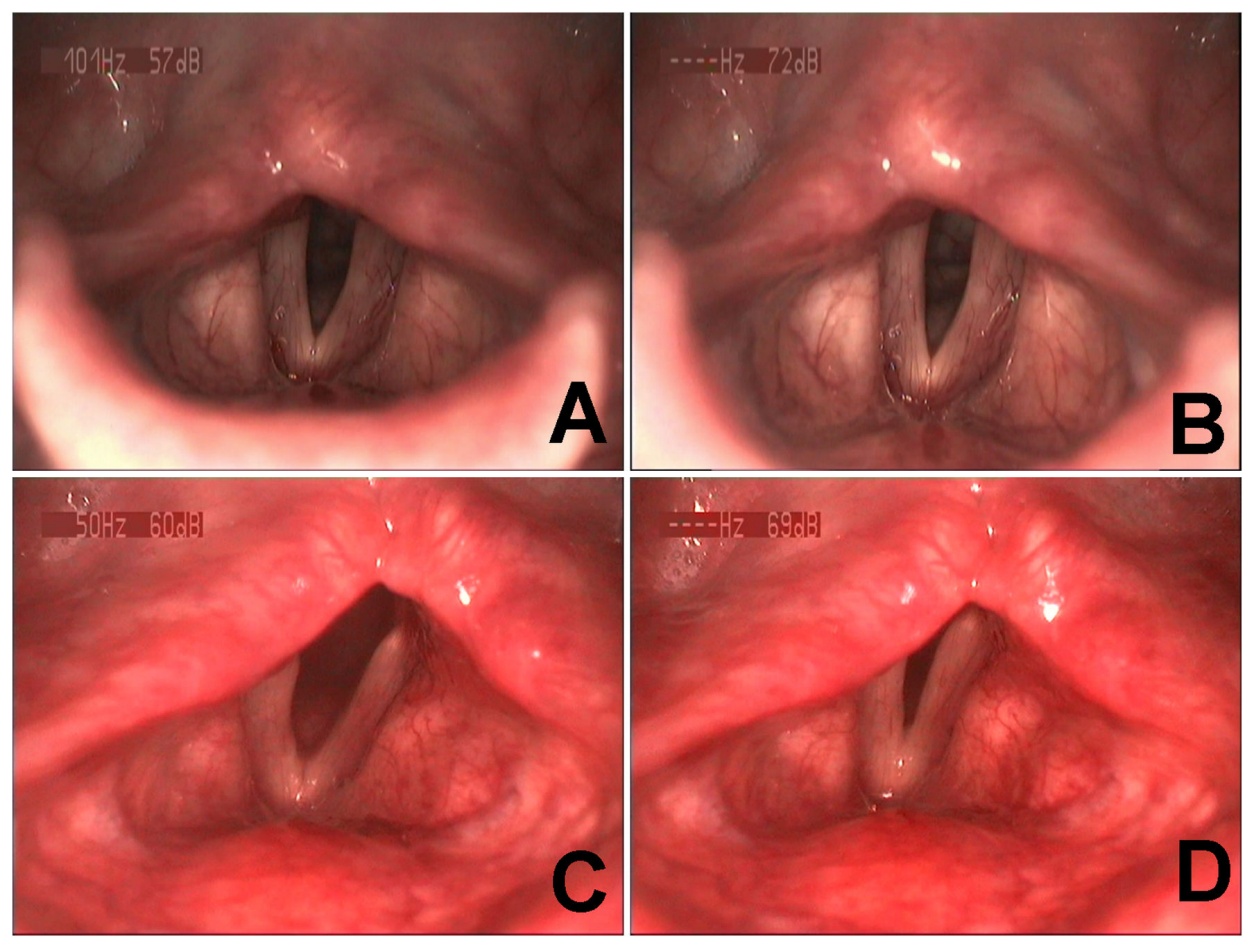
Preoperative and postoperative videostroboscopic findings. Case No. 5 showed representative bilateral vocal fold paralysis. Preoperatively, the bilateral vocal folds were fixed in the paramedian position during inspiration (A). Severely incomplete glottic closure occurred during phonation (B). Six months postoperatively, the bilateral vocal folds abducted to an paramedian or intermediate position during inspiration (C) and adducted back to the near-midline position during phonation with moderate incomplete glottis closure (D).

The preoperative and postoperative laryngeal appearance of the patients are shown in Table 1. Preoperative video recordings (see Video S1) showed that most vocal folds were fixed in the paramedian or midline position, with varying glottic chink during inspiration (Figure 2A), and with incomplete or complete glottal closure during phonation (Figure 2B). In addition, most vocal folds showed straight or mildly bowing edges with no significant difference between the left and right sides (*P* > 0.05). Two to 6 months after reinnervation surgery, video recordings (see Video S2) showed variable excursion of abductive movement during inspiration in the left vocal folds of 87% of patients and the right vocal folds of 72% of patients (Figure 2C). The excursion of abductive movement increased to varying degrees after physical activity, and the excursion of abductive movement on the left side was significantly larger than that on the right side (*P* < 0.05). The vocal folds were in the paramedian or midline position during phonation, with varying degrees of glottic chink (Figure 2D), but without a significant difference compared with the preoperative situation (*P* > 0.05) (Table 1). Three cases presented immobile vocal folds during inspiration and phonation, another 3 cases recovered evident vocal fold abduction during inspiration in early follow-up postoperatively, however, vocal fold adduction during inspiration (paradoxical movement) was observed in later follow-up, these 6 patients were defined as failure cases.

**Table 1:** Comparison of pre- and post-operative videostroboscopic findings.

**Rating Rank**	**No. of videostroboscopic findings**		**P value**
	Preoperative	Postoperative	
**Glottal closure during phonation**			0.9314
**Complete**	13	11	
**Slightly incomplete**	11	13	
**Moderately incomplete**	14	13	
**Severely incomplete**	6	7	
**Functional abduction of left vocal fold**			P<0.001
**No abduction**	44	6	
**Slight abduction**	0	9	
**Moderate abduction**	0	19	
**Severe abduction**	0	10	
**Functional abduction of right vocal fold**			P<0.001
**No abduction**	44	12	
**Slight abduction**	0	19	
**Moderate abduction**	0	9	
**Severe abduction**	0	4	
**Edge of left vocal fold**			0.7234
**Straight**	22	17	
**Mildly bowing**	13	15	
**Moderately bowing**	8	10	
**Severely bowing**	1	2	
**Edge of right vocal fold**			0.5607
**Straight**	23	18	
**Mildly bowing**	15	19	
**Moderately bowing**	6	6	
**Severely bowing**	0	1	

### Vocal function assessment

Preoperative and postoperative voice samples were available from all 44 patients for perceptual evaluation and acoustic analysis. There were no significant differences in the overall grade, roughness, breathiness, asthenia, and strain between the preoperative and postoperative values (*P* > 0.05) (see Table 2). Postoperative jitter, shimmer were lower, while NHR was higher than the corresponding preoperative values, but without a significant difference (*P* > 0.05) (see Table 3). Postoperative MPT values were longer than the preoperative values, but still without a significant difference (*P* > 0.05) (see Table 3).

**Table 2:** Pre- and post-operative perceptual evaluation.

**Parameter**	**N**	**Preoperative**	**Postoperative**	**P value**
		Median(Q_L_, Q_U_)	Median(Q_L_, Q_U_)	
**Grade**	44	1.6 (1.2,2.1)	1.6 (1.4,1.8)	0.5955
**Roughness**	44	1.6 (1.2,1.8)	1.6 (1.4,1.7)	0.9034
**Breathiness**	44	1.7 (1.4,2.0)	1.6 (1.6,1.8)	0.5576
**Asthenia**	44	1.6 (1.3,2.0)	1.6 (1.4,1.8)	0.8277
**Strain**	44	1.0 (1.0,1.2)	1.2 (1.0,1.4)	0.0740

Q_L_=low quartile, Q_U_=upper quartile

**Table 3:** Pre- and post-operative vocal function assessment.

**Parameter**	**N**	**Data of vocal function assessment ($\bar{x}\pm s$)**		**T**	**P value**
		Preoperative	Postoperative		
**Jitter**	44	1.19±0.54	1.07±0.36	1.38	0.175
**Shimmer**	44	7.92±2.33	7.19±1.71	1.96	0.057
**NHR**	44	0.08±0.04	0.09±0.14	0.53	0.599
**MPT**	44	8.93±3.22	9.21±1.61	0.61	0.543

NHR = mean noise to harmonics ratio, MPT = maximum phonation time

### Pulmonary function test

During the follow-up examinations, no patient was found to have breathing problems or marked breathing restrictions during physical activity immediately after the operation. Chest X-ray examination revealed diaphragmatic paralysis and elevation on the left side immediately after cutting the phrenic nerve in those patients who had no accessory phrenic nerves. Excursion of diaphragmatic motion recovered to 22–31%, 35–76%, and 40–82% of the preoperative motion 3, 6, and 12 months after the surgery, respectively. Because VC, FVC, FEV1, and PImax are affected by upper airway obstruction, six patients in whom functional vocal fold abduction was not achieved were excluded from the analysis of pulmonary function. The average VC, FVC, FEV1, and PImax values 3 months postoperatively were significantly decreased compared with the preoperative values and normal reference values (*P* < 0.05). All parameters with the exception of FVC were still decreased with a significant difference compared with normal reference values 6 months after surgery (P < 0.05); However, All parameters with the exception of PImax were significantly higher than preoperative values 6 months after surgery (*P* < 0.05); All parameters with the exception of PImax recovered to normal reference value levels within 12 months after surgery. PImax decreased significantly compared with the normal reference values 1 year postoperatively, but remained higher than the preoperative values (*P* < 0.05) (see Table 4).

**Table 4:** Pre- and post-operative pulmonary function parameters.

**Parameter**	**N**	**Data of pulmonary functional parameters（$\bar{x}\pm s$)**				
		Normal Reference	Preoperative	3 months post-	6 months post-	12months post-
**VC**	38	3.29±0.82	2.64±0.71	2.49±0.80	3.19±0.83	3.30±0.84
**FVC**	38	3.29±0.82	2.68±0.74	2.37±0.80	3.20±0.75	3.25±0.80
**FEV1**	38	2.78±0.71	2.29±0.66	2.18±0.63	2.68±0.64	2.78±0.69
**PI_max_**	38	88.79±11.50	62.39±14.89	41.32±8.96	54.39±11.20	66.13±11.29

VC: Vital Capacity; FVC: Forced vital capacity; FEV1: Forced expiratory volume in 1 second; PI max: Maximal inspiratory pressure; Post- Post-operative

### EMG findings

Preoperative and postoperative EMG results were both available in 31 patients. The electrical activity during the EMG was divided into two types: spontaneous activity and MUR. Preoperatively, abnormal spontaneous activities such as positive waves, fibrillations, or complex repetitive discharges were recorded in the PCA muscle and TA muscle of the left side in 29.0% (9/31) and 25.8% (8/31) of patients, respectively, and in the PCA muscle and TA muscle of the right side in 32.3% (10/31) and 22.6% (7/31) of patients, respectively. One year after surgery, there was no evidence of abnormal spontaneous activities in the PCA muscles of both sides at rest, but abnormal spontaneous activities were recorded in the left TA muscle in 25.8% (8/31) of patients and in the right TA muscle in 22.6% (7/31) of patients. Preoperative and postoperative MUR of bilateral PCA muscles during deep inspiration and MUR of bilateral TA muscles during phonation are shown in Table 5. The results indicate significant improvement in postoperative MUR compared with preoperative MUR in the PCA muscle during deep inspiration on both sides (*P* < 0.05). In addition, the postoperative MUR in the PCA muscles was significantly stronger on the left side than on the right side (*P* < 0.05); however, postoperative MUR in bilateral TA muscles during phonation was represented mostly as simple interference or no motor unit potential and did not show any significant improvement compared with the preoperative situation (*P* > 0.05). Misdirected regeneration motor unit potential was recorded in the TA muscles during deep inspiration in four of the failed patients, but in none of the PCA muscles postoperatively.

**Table 5:** Pre- and post-operative MUR in laryngeal muscles.

**MUR**	**No. of left side (n=31)**				**No. of right side (n=31)**			
	Preoperative		Postoperative		Preoperative		Postoperative	
	PCA	TA	PCA	TA	PCA	TA	PCA	TA
**Full interference**	1	2	26	0	1	1	18	0
**Mixed interference**	6	8	5	3	5	6	13	2
**Simple interference**	16	15	0	25	18	19	0	25
**No MUP**	8	6	0	3	7	5	0	4

MUR: Motor-unit recruitment; PCA: Posterior cricoarytenoid muscle; TA: Thyroarytenoid muscle; MUP: Motor-unit potential

## Discussion

Management of BVFP is a substantial challenge to laryngologists. Traditional treatments such as arytenoidectomy, which mainly widens the airway at the glottis level, may worsen the voice and lead to aspiration, greatly compromising the quality of life of patients. An optimal treatment should strike a balance between airway and voice, which could be theoretically attained by selective reinnervation of the PCA muscle [17].

Direct anastomosis of the two severed stumps of the RLN has been studied. This technique may reportedly achieve reinnervation of the laryngeal muscles; however, the vocal folds were fixed at the midline or paramedian position, or paradoxical movement of the vocal cords was observed due to misdirected regeneration of the adductor and abductor fibers, which is also called laryngeal synkinesis [18]. Later, a donor nerve such as the *ansa cervicalis*, vagus nerve, or phrenic nerve was anastomosed to the distal stump of the RLN for reinnervation of the PCA muscle. However, the results were disappointing because both the adductor and abductor muscles are reinnervated by the same nerve, so the vocal folds remain fixed in one position or show paradoxical motion. Many later researchers stated that selective reinnervation of the PCA muscles may help to avoid laryngeal synkinesis [5-7,11,17]. However, the abductor branches of the RLN are usually fairly small in diameter, and several branches which innervate the PCA muscle commonly originate from the RLN trunk. This makes direct neurorrhaphy technically challenging, even under surgical magnification. NMP implantation into the PCA muscles at one time seemed to have reached the goal of selective reinnervation of the PCA muscles. It has been used for treatment of BVFP in animal models [1,3,4,19] and has been clinically applied in humans [11,20]; however, the results were inconsistent and unpredictable. Similar results were also reported in direct implantation of the donor nerve into the PCA muscle [5,21].

The phrenic nerve is considered to be the ideal donor nerve for reinnervation of the PCA muscles from histological, anatomical, and physiologic viewpoints. Methods of reinnervating the PCA muscle using the phrenic nerve as reported in the literature include at least: 1) anastomosis of the phrenic nerve to the abductor branch of the RLN, 2) anastomosis of the phrenic nerve to the RLN with the adductor branches of the RLN severed, 3) implantation of the split phrenic nerve into the PCA muscle belly, 4) implantation of the phrenic nerve trunk into the PCA muscle belly, 5) anastomosis of the phrenic nerve to the RLN with anastomosis of the ipsilateral severed adductor branch of the RLN to the *ansa cervicalis* or other donor nerves, and 6) anastomosis of the phrenic nerve to one side of the RLN with anastomosis of the severed adductor trunk of the RLN to the contralateral RLN to achieve bilateral PCA muscles reinnervation. Most of the above studies were limited to animal experiments, in most of which restoration of evident vocal fold abduction was reported. In one of our previous animal studies, we anastomosed the upper branch of the phrenic nerve to the RLN, and at the same time, the adductor branches of the RLN were severed and implanted into the PCA muscle. We assumed that with this method, all regenerative phrenic nerve fibers would grow into the PCA muscle instead of wasting some in the ligated stump of the adductor trunk. Thus, two different sets of motor end plates would be established, which is termed “double reinnervation” [7]. This is in agreement with the point of view of Kwak, who stated that insertion of the proximal stump of the adductor branch of the RLN into the PCA muscle not only allows for twice the stimulation of the muscle to achieve abduction, but also prevents aberrant reinnervation of the adductor muscles by the abductor fibers [22]. Our results showed that all animals restored vocal fold abduction, and abduction excursion of some vocal folds even exceeded normal ones [7], indicating that this procedure is an effective method to reinnervate the PCA muscle. We treated six patients with BVFP using this surgical technique in clinical practice, and promising results were obtained in five of them. We modified the above-described technique by using a free nerve graft that was harvested from the cervical plexus for bridging between the adductor trunk of the left RLN and trunk of the right RLN. Our results in animal models using this modified technique showed satisfactory restoration of reinnervation of the bilateral PCA muscles by a single phrenic nerve (unpublished data), which is in accordance with the results reported by Baldissera et al [23]. Encouraged by these promising results, we applied this modified technique clinically in the present study.

Videostroboscopic, voice analysis and laryngeal electromyographic data confirmed the success of the modified technique. The evidence is as follows: 1) Inspiratory vocal fold abduction was restored on both sides, and abductive excursion increased after physical activity. 2) We successfully reinnervated the bilateral PCA muscles with a single phrenic nerve; the success rate of abductor reinnervation was 87%. 3) Voice function assessment showed that the voice of some patients improved after reinnervation surgery, although the results showed no significant difference. 4) Electromyographic examination showed typical inspiratory high-frequency discharge in the PCA muscles on both sides. 5) Resection of the left phrenic nerve was associated with no immediate clinical symptoms, such as airway compromise. Pulmonary function test results confirmed no long-term adverse effects on pulmonary function.

One phenomenon observed in this study is that abductive excursion of the left vocal fold was larger than that of the right side, and the recovery rate of vocal fold abduction on the left side was higher than that on the right side. We presume that this was partly due to the number of regenerative axons that pass through the anastomotic site. In the present study, we chose the left phrenic nerve as the donor nerve, which was transected and anastomosed to the distal stump of the left RLN trunk. The adductor branches were then cut, and their common trunk was anastomosed to a free nerve graft whose other end was carried contralaterally and sutured to the distal end of the right RLN trunk. The phrenic nerve fibers needed to pass through one anastomotic site to reach the left PCA muscle, but through three sites to reach the right PCA muscle. Some of the available regenerated axons were lost across each anastomotic site; thus, reinnervation procedures that involve an interposition nerve graft with two anastomoses are less effective [24]. However, our results showed that the right PCA could still gain adequate or partial reinnervation to restore abduction of the right vocal fold even when regenerative axons had to pass through three anastomotic sites; the recovery rate of vocal fold abduction was 70%. This indicates that satisfactory bilateral abduction movement can be achieved in humans by reinnervating the bilateral PCA muscles using a single phrenic nerve.

The voice quality of patients in this series did not worsen as did the voice quality of those who underwent arytenoidectomy. Interestingly, although we did not intentionally perform adductor reinnervation in the present study, we still observed evident vocal fold adduction during phonation, and some patients showed complete glottis closure. The reasons may be as follows: 1) The vocal folds passively adducted after abduction motion. 2) Laryngeal EMG showed that the motor unit potential was recorded in the TA muscles, which indicates subclinical reinnervation of the adductor muscles due to axonal regeneration across the transected distal ends of adductor branches of the RLN into the adductor muscles or neurotization that sprouted from the surrounding musculature, which may have arisen from the superior laryngeal nerve or from the nerve that innervates the cricopharyngeal muscle [9,18,25]. 3) Contraction of the uninjured cricothyroid muscle may also contribute to the adduction of the vocal folds [18].

Phrenic nerve transfer has been used to repair brachial plexus nerve injuries, and many researchers have studied pulmonary function following unilateral phrenic nerve transection. Fackler et al. reported 14 patients who underwent unilateral phrenic nerve transection; VC returned to the preoperative normal level in all patients 6 months after surgery [26]. Xu et al. reported a series of cases involving phrenic nerve neurotization and showed that the pulmonary functional parameters FVC and FEV1 were not affected by the procedure in the long term [27]. Luedemann et al. reported a series of 23 patients who underwent neurotization surgery with the phrenic nerve as the donor nerve and found that none of the 23 patients experienced pulmonary problems postoperatively; however, the patients incurred a higher risk of reduced pulmonary VC when using the right phrenic nerve than the left one. The authors suggested that if possible, the left phrenic nerve should be used [28]. Based on the above results and those of our previous report [11], we used the left phrenic nerve for transfer in all patients in the present study; none of them experienced pulmonary problems following the surgery. Pulmonary functional parameters indicated mild reduction in pulmonary function 3 months and 6 months postoperatively; however, all parameters—with the exception of PImax—recovered to normal reference value levels by 12 months postoperatively. A decrease in PImax is not an absolute indication of pulmonary function reduction because PImax is affected not only by the strength of the respiratory muscles, but also by the size of the glottic chink [29]. The absence of PImax recovery to the normal reference value in the present study may have been due to the glottic chink not recovering its normal size. However, the preoperative general condition of the patients is also very important. Pulmonary functional tests and chest X-rays must be performed to evaluate the preoperative diaphragm motion and exclude pulmonary diseases such as chest or lung trauma, emphysema, and chronic obstructive pulmonary disease.

There are several critical influencing factors which may affect the surgical outcome of phrenic nerve transfer surgery. The first important factor that may have influenced the outcome of surgery is the interval between the phrenic nerve transfer procedure and the onset of bilateral RLN injury. Theoretically, better results of reinnervation surgery are obtained if the interval between the injury and reinnervation is shorter, as is shown both in the literature and in our experimental study [6,30]. We performed the phrenic nerve transfer surgery for some BVFP patients with thyroid surgery-related RLN injury within 6 months after the injury, there are some reasons. Firstly, we performed exploratory surgery for BVFP patients whose denervation duration was less than 6 months only when the surgeons of primary thyroid surgery strongly suggested that bilateral RLNs were probably transected. Actually, bilateral RLNs were confirmed to be completely transected during exploratory surgery in 18 BVFP patients, and phrenic nerve transfer surgery was then performed immediately. However, when RLN exploration confirmed RLN continuity (not transected as suggested by the original thyroid surgeons), then nerve decompression surgery was performed (These patients were not included in the present study). Some of these patients did indeed benefit from the decompression surgery and their vocal folds regained physiologic vocal fold movement after decompression. This was consistent with some previous reports [31-34]. Secondly, progressive atrophy with denervation time may impair the regenerative capacity of skeletal muscles owing to irreversible pathologic alterations, such as muscle fibrosis and degeneration of muscle endplates [6]. If bilateral RLNs are completely transected, the degree of subclinical reinnervation is less, the atrophy of PCA muscles is more severe. Thirdly, as the only one abductor muscle of intrinsic laryngeal muscles, PCA muscle plays an important role in the process of respiration. Study on denervated laryngeal muscles of rats showed that denervation produced large shifts in the expression of the fast type IIX (ie, up-regulated) and IIB (ie, down-regulated) myosin heavy chain (MyHC) isoforms in the PCA muscle. However, reinnervation of the PCA muscle had little effect on reversing these shifts at the whole muscle level, in contrast, reinnervation of the TA muscle was highly effective in restoring a normal pattern of MyHC isoform expression at the whole-muscle level [35]. Consistent with these observations, Shiotani et al also reported that repair of the recurrent laryngeal nerve did not prevent denervation-like shifts in the MyHC isoform profile of the PCA muscle but was effective in minimizing shifts in the MyHC isoform profile in TA muscle [36]. Our research on denervated PCA muscle on humans also demonstrated that although type I MyHC isoforms and type II MyHC isoforms changed significantly with the denervation time increases, the prominent transitions in MyHC isoforms expression take place one year after RLN injury (unpublished data). Last but not least, in this series, three patients whose interval time between bilateral RLN injury and reinnervation surgery was >10 months did not show restoration of abduction in the follow-up, which strongly indicates that it is better to perform phrenic nerve transfer surgery for patients with BVFP whose dennervation course is <10 months. This is totally different from the situation in UVFP, in which reinnervation surgery could still be effective with denervation duration of several years. However, within 10 months after bilateral RLN injury, when is the better time range to perform phrenic nerve transfer surgery so as to achieve the most effectiveness needs further investigation, which will depend on further accumulation of cases and multivariate analysis on the influencing factors of the surgical procedure. In summary, the above findings indicate that early reinnervation surgery should be performed for BVFP patients if bilateral RLNs were confirmed to have been transected. However, the prognostic value of laryngeal PCA muscle EMG for BVFP is limited and sometimes not precise. So the most direct means to confirm the RLN transection (in our experience) is exploratory surgery in the first few months after RLN injury when surgeons of the primary thyroid surgery suggested that RLNs might have been transected. Our experience appears to show that RLN decompression can be helpful in patients in whom the RLNs have been ligated, but these data are not included in the present study. The authors concede that it is conceivable that we may have over-treated those patients by exploratory surgery during the early stage of RLN injury in a very small proportion of patients who might possibly have subsequently demonstrated spontaneous recovery of physiological vocal fold motion without any intervention. However, for most BVFP patients whose bilateral RLNs were indeed transected, they could benefit from early intervention because physiologic vocal fold abduction could be achieved if phrenic nerve transfer surgery were performed in early stage.

Patient age is another critical factor. All patients in this series were younger than 55 years with the exception of one, who was 59 years old at the time of the surgery and insisted on undergoing phrenic nerve transfer surgery. We informed the patient the possible ineffective outcome. The procedure was performed in this case after she signed the consent inform. Follow-up examinations showed no inspiratory vocal fold abduction. Considering the results of our previous report [11], we assume that the adverse surgical outcome was to some extent related to the patient’s older age. However, because of the small sample of this study, we don’t feel that it is conclusive to say that old age (>55 years old) leads to adverse outcomes of reinnervation surgery.

Based on our experience, the following surgical aspects of this procedure may help to improve the outcome. 1) It is critical to identify the interarytenoid branch of the RLN, thoroughly transect it and ligate its proximal end. The interarytenoid branch usually originates from the genu segment of the RLN just below the level of the cricoarytenoid joint. There are usually one to several abductor branches below the interarytenoid branch, which are also much smaller in diameter. Videostroboscopy showed immobile vocal folds in 3 cases, and abduction of vocal folds observed during early follow-up turned into paradoxical motion during later follow-up in another 3 patients. If the interarytenoid branch was not precisely identified and thoroughly cut or its proximal end was not ligated during the surgery, regenerative abductor fibers may regrow into the interarytenoid muscle through the distal end of the severed interarytenoid branch, or even sprout into other adductor muscles so as to result in laryngeal synkinesis, which most likely led to the failure of the surgery. 2) To expose and identify the interarytenoid branch, a small part of cartilage must be removed from the posteroinferior part of the thyroid lamina; however, the integrity of the superior and inferior process of the thyroid cartilage should be maintained. 3) The nerve supply pattern of the human PCA muscles is usually variable and complex and is present as two or more branches [1]. Care should be taken to avoid injuring the abductor muscle branches of the RLN. 4) The free nerve graft, which is usually harvested from one branch of the cervical plexus, should be comparable with the RLN in diameter and sufficiently long to achieve tension-free anastomosis. The length of the free nerve graft is not problematic if passed contralaterally through a retrolaryngeal or retrotracheal tunnel. 5) The nerve ends of the RLN distal stumps should be prepared by thorough resection of scarred or nonviable tissue to expose the normal nerve fascicles. 6) It is important to avoid injury of the cricoarytenoid joint and evaluate arytenoid mobility before and during surgery. 7) The left phrenic nerve should be used preferentially in order to avoid a higher risk of reduced pulmonary function.

Theoretically, the ideal treatment for BVFP is restoration of physiologic (bidirectional) motion of the bilateral vocal folds, including phasic abduction during inspiration, volitional closure for phonation, and ideally, a glottic closure reflex during swallowing or as a response to sensory stimuli [37]. Many researchers have studied combinations of adductor and abductor reinnervation techniques using animal models, and some reported restoration of physiologic motion of the vocal folds [38,39]. Marie clinically applied a combined reinnervation technique in 12 patients with BVFP, and only three of six evaluable patients achieved promising results. This success rate is not very satisfactory [12]. Other experimental studies reported good adduction, but no or poor abduction [40,41]. Crumley and Marie reported the results of animal experiments in which the abductor muscles were reinnervated by the phrenic nerve and the adductor muscles were reinnervated by the *ansa cervicalis* simultaneously. Coordinated vocal fold movements were observed in only a few of the animals. The reason may be that regenerative axons from the *ansa cervicalis* reach the abductor muscle before those from the phrenic nerve, so they may reinnervate the PCA muscle by sprouting into it from the *ansa cervicalis* anastomostic site [41]. This phenomenon was also evident in our previous animal experiments. Therefore, we preferentially studied the restoration of vocal fold abduction in the present study. The promising results of PCA muscle reinnervation in this pioneer study pave the path for our further work on combinations of reinnervation of both abductor and adductor muscles.

## Conclusions

Reinnervation of the bilateral PCA muscles using the left phrenic nerve restored inspiratory vocal fold abduction to a satisfactory extent while preserving phonatory function at a preoperative level. None of our patients developed immediate dyspnea after surgery, and the pulmonary function parameters were recovered to normal reference value levels within 1 year, indicating the safety of this procedure.

## Supporting Information

Video S1 **Preoperative videostroboscopic finding of a BVFP patient whose denervation course was 8 months.** The preoperative video recording showed that the bilateral vocal folds were fixed in midline position with a small glottic chink during inspiration. Complete glottis closure occurred during phonation.
(MP4)

Video S2 **Six month postoperatively, the bilateral vocal folds abducted to an intermediate or lateral position during inspiration, the excursion of abduction movement on the left side was larger than that on the right side, and the bilateral vocal folds adducted back to near-midline position during phonation, with severely incomplete glottis closure.**
(MP4)
